# Coal Fly Ash Impairs Airway Antimicrobial Peptides and Increases Bacterial Growth

**DOI:** 10.1371/journal.pone.0057673

**Published:** 2013-02-28

**Authors:** Jennifer A. Borcherding, Haihan Chen, Juan C. Caraballo, Jonas Baltrusaitis, Alejandro A. Pezzulo, Joseph Zabner, Vicki H. Grassian, Alejandro P. Comellas

**Affiliations:** 1 Department of Internal Medicine, Carver College of Medicine, University of Iowa, Iowa City, Iowa, United States of America; 2 Department of Chemistry, University of Iowa, Iowa City, Iowa, United States of America; 3 Department of Chemical and Biochemical Engineering, Iowa City, Iowa, United States of America; 4 Central Microscopy Research Facility, University of Iowa, Iowa City, Iowa, United States of America; Institut de Pharmacologie et de Biologie Structurale, France

## Abstract

Air pollution is a risk factor for respiratory infections, and one of its main components is particulate matter (PM), which is comprised of a number of particles that contain iron, such as coal fly ash (CFA). Since free iron concentrations are extremely low in airway surface liquid (ASL), we hypothesize that CFA impairs antimicrobial peptides (AMP) function and can be a source of iron to bacteria. We tested this hypothesis *in vivo* by instilling mice with *Pseudomonas aeruginosa* (PA01) and CFA and determine the percentage of bacterial clearance. In addition, we tested bacterial clearance in cell culture by exposing primary human airway epithelial cells to PA01 and CFA and determining the AMP activity and bacterial growth *in vitro*. We report that CFA is a bioavailable source of iron for bacteria. We show that CFA interferes with bacterial clearance *in vivo* and in primary human airway epithelial cultures. Also, we demonstrate that CFA inhibits AMP activity *in vitro*, which we propose as a mechanism of our cell culture and *in vivo* results. Furthermore, PA01 uses CFA as an iron source with a direct correlation between CFA iron dissolution and bacterial growth. CFA concentrations used are very relevant to human daily exposures, thus posing a potential public health risk for susceptible subjects. Although CFA provides a source of bioavailable iron for bacteria, not all CFA particles have the same biological effects, and their propensity for iron dissolution is an important factor. CFA impairs lung innate immune mechanisms of bacterial clearance, specifically AMP activity. We expect that identifying the PM mechanisms of respiratory infections will translate into public health policies aimed at controlling, not only concentration of PM exposure, but physicochemical characteristics that will potentially cause respiratory infections in susceptible individuals and populations.

## Introduction

Coal is one of the most abundant sources of energy production globally and continues to grow on an annual basis. In 2010, U.S. coal consumption was 1,048.3 million short tons, an increase of 50.8 short tons from the previous year [1]. Coal Fly Ash (CFA), a byproduct of coal combustion, is considered a poorly soluble particle comprised of various transition metals such as iron, and aluminum silicate as classified by ACGIH (American Conference of Industrial Hygienists) [2]. The majority of CFA (99%) is collected and deposited in landfills, therefore providing a potential source of transition metals into the water supply and redistributing itself into the atmosphere [3]. Due to the increased global demand and the limited regulations in growing economies such as China, CFA released into the atmosphere continues to be a large anthropogenic source of air pollution.

Epidemiological studies show a strong correlation between respiratory infections and PM_2.5_ [4]. Ambient air pollution is associated with cystic fibrosis (CF) and chronic obstructive pulmonary disease (COPD) exacerbations [4], [5], [6]. The majority of these exacerbations are infectious in nature [7]. In addition, a correlation between biomass fuels used for indoor cooking, including coal, and acute respiratory infections in children has been reported [8]. Therefore, due to the association between respiratory exacerbations and increased pollution, further investigation needs to be conducted in order to understand the mechanism of PM induced respiratory infections.

PM which is rich in iron [9] can increase iron bioavailability to microorganisms [10], [11], such as *Pseudomonas Aeruginosa* (PA01). The amount of soluble, and therefore potentially bioavailable iron in PM, specifically CFA, has been correlated with particle size [12], source of CFA [12] and amount of aluminum silicate present within the particle [13]. Therefore, CFA can be an exogenous iron source for bacteria in biological fluids, such as the airway surface liquid (ASL), that the body maintains at low iron concentrations (<10^−18^ M) [14] and thus become potentially detrimental to human health. Although there have been significant studies of the effects of PM on the lung epithelium, there is a paucity of data on the effects of PM induced bacterial growth and pathogenicity that can lead to respiratory infections.

We hypothesize that CFA will impair airway bacterial clearance by both promoting bacterial growth and impairing airway epithelial antimicrobial peptide function. To test this hypothesis we set out to determine the effects of CFA on *Pseudomonas Aeruginosa* (PA01) *in vivo* and *in vitro*. Three CFA particles with different iron content, that were previously characterized for iron solubility and mobilization, were used for this study (Table 1) [11].

**Table 1:** Coal Fly Ash Particles.

Total Iron Content, Dissolved Iron %, and Elemental Composition of Coal Fly Ash [11]			
	FA 2689	FA 2690	FA 2691
**Source**	Stilesboro, GA	Criag, CO	Iatan, MO
**Size**	1–50 µm	1–50 µm	1–50 µm
**Specific Surface Area (m^2^** **g^−1^)**	0.8±0.1	3.8±0.1	2.2±0.1
**Total Fe Content (%)**	9.32±0.06	3.57±0.06	4.42±0.03
**Dissolved Fe (%) pH 7.5**	0.028	0.032	0.057
**XPS/EDX Fe** a	1.3±0.2	1.4±0.3	0.6±0.2
**XPS/EDX Al** a	0.6±0.1	0.6±0.1	0.4±0.2
**XPS/EDX Si** a	1.0±0.1	0.7±0.1	0.7±0.3

Total iron content, aluminum silicate content, dissolved iron, particle size, and specific surface area of three different coal fly ash particles (FA 2689, FA 2690 and FA 2691).

XPS was used to determine surface composition.

EDX was used to determine bulk composition.

a XPS/EDX Ratio: High ratio of XPS/EDX indicates elemental enrichment at the surface, low ratio content (<1) indicates enrichment of element at inner core.

## Methods

### Ethics Statement

All animals (mice) used in this study were according to protocols approved by the University of Iowa Institutional Animal Care and Use Committee (IACUC). Animals were anesthetized prior to instillations and harvest in order to reduce animal distress.

### X-Ray Photoelectron Spectroscopy (XPS)

Surface composition for all fly ash particles was performed using a custom-designed Kratos Axis Ultra X-Ray photoelectron spectrometer with a monochromatic Al Ka X-Ray source as previously described [15]. The fly ash particles were pressed onto indium foil which was fixed on a stainless steel bar or copper stub for further analysis. The pressed particles were then transferred into the XPS analysis chamber, which had a pressure that was maintained in the 10^−9^ Torr range during analysis. Charging was prevented by using the following instrumental parameters: energy range from 1200 to −5 eV, pass energy of 160 eV, step size of 1 eV, dwell time of 200 ms, and an X-ray spot size of ∼700×300 µm. Survey spectra were collected at three different locations on the sample stub, and reported elemental compositions represent the average and one standard deviation of the three analyses. XPS data collected were analyzed using CasaXPS data processing software.

### Energy Dispersive X-Ray Spectroscopy (EDX)

The morphology and total elemental composition of fly ash particles were examined using a Hitachi S-3400 N scanning electron microscopy (SEM) coupled with energy dispersive X-ray spectroscopy system. Particles were sprinkled onto carbon tape that had been mounted on an aluminum stub and were subsequently carbon coated. Elemental analyses used an integrated IXRF System Inc. X-ray microanalysis system and an accelerating voltage of 10 kV with a detection limit of 1 wt%. SEM/EDX elemental maps were collected as well to examine the distribution of Fe in fly ash particles. A resolution of 256×200 pixels, and a dwell time of 1 second were used.

### Experimental Preparation of Particles

Characterized coal fly ash from the National Institute of Standards and Technology (NIST) were suspended in an iron deficient media [BD Difco Minimal media (M9) with 2.2 mM glucose, 0.002 M magnesium sulfate (MgSO_4_), 0.001 M calcium chloride (CaCl_2_) and 25 mM sodium succinate]. The particle suspension was sonicated for ten minutes immediately prior to conducting experiments.

### Bacteria

*Pseudomonas Aeruginosa* (PA01) was chosen as a model in our studies due to its prevalence and importance in disease such as COPD and CF.

### *In Vivo* Mouse Instillation

Six to eight week Harlan C57/BL6 males (20–25 g) were intranasally instilled with 50 µl OD_600_ = 0.03 PA01with or without 10 µg/mL freshly dissolved and sonicated CFA. PA01 was exposed to CFA for a minimal amount of time (∼10 minutes). After 24 hours, BAL was performed or lungs were removed and homogenized in 2 ml PBS. In BAL performed mice, BALF samples were used to determine cell count and differential by using Wright-Geisma staining. Non-lavaged samples were plated on lauria broth agar (LB) plates and CFUs of PA01 were recorded.

### TNF-α and IL- 1β

R& D DuoSet ELISAs were conducted according to manufacturer’s instructions to determine TNF-α and IL-1β production in BAL fluid.

### Cell Culture

Briefly cells were isolated from donor lungs and plated on cell culture inserts in an air liquid interface. Human airway epithelial cells were obtained from the University of Iowa cell culture core and changed to antibiotic free USG media two weeks prior to experiments [16]. Cells were washed with PBS three times and media was changed to antibiotic free USG media two weeks prior to experiments. Media was changed every four days and experiments were conducted day four post media change in order to ensure adequate airway surface liquid levels. Sterility of cell culture was determined as previously published (Phil H. Karp 2002). Briefly, a dose response of PA01was conducted to determine inoculum of complete bacterial killing. 0.1 µL with 12 CFU PA01 and 10 µg/mL CFA was placed on apical surface. Growth was determined by washing epithelial apical surface with 50 ul PBS and growing in LB media for eight hours.

### p-hydroxyphenylacetate Assay (pHPA)

H_2_O_2_ was measured by adding 1.6 mM pHPA (Sigma), 95 µg/mL Horseradish Peroxidase (HRP) (Sigma), 1 mM Hepes, 6.5 mM glucose and 6 mM NHCO_3_ in Hanks balanced salt solution (HBSS). Solution was added to cell culture and fluorescence was measured over one hour. A standard H_2_O_2_ curve was generated and pHPA dimer concentration was determined.

### Transepithelial Electrical Conductance (Gt)

Airway cells were submerged in 500 µl of media and transepithelial electrical resistance (*Rt*) was measured with Millicell Electrical Resistance System (ERS) (Millipore Corporation, Bedford, MA) and *Gt* was calculated as the reciprocal of *Rt* [17].

### Antimicrobial Peptide Activity

PA01 was grown overnight in M9 media, subcultured and diluted to OD_600_ = 0.45. The culture was then diluted and 13500 PA01 was added to start experiment. Sodium phosphate buffer at pH 7.8 was used and a cocktail of antimicrobial peptides (600 µg/mL Lysozyme, 200 µg/mL Lactoferrin and 100 ng/mL β-Defensins 1&2) equaling 400 µl were added to a 96 deep well plate. 10 µg/mL CFA was added with AMPs and PA01. Mixture was incubated for one hour at 37°C and 300 rpm. ¼ diluted Lauria Broth (LB) media was added to mixture and grown overnight. OD_600_ was measured to determine level of antimicrobial peptide activity. CFUs were determined by conducting the above experiment, serially diluting and plating cultures on LB agar plates at beginning and endpoints to determine exact colony count.

### Growth Experiments

PA01 was grown overnight in an iron deficient media BD Difco Minimal media (M9) with 2.2 mM glucose, 0.002 M magnesium sulfate (MgSO_4_), 0.001 M calcium chloride (CaCl_2_) and 25 mM sodium succinate. 10 µg/mL CFA was added to three hour sub-cultured overnight cultures to equal a volume of 250 µl and growth was observed by measuring OD_600_ at 37°C for nine hours. 25 µM iron chloride (FeCl_3_) [18], a soluble source of iron was used as a positive control. CFU experiments were conducted in a 5 mL volume in same conditions as above. Samples were taken at time T = 0 and T = 18, serially diluted and plated on LB plates to determine exact colony count.

### Fe-Dissolution

An inductively coupled plasma optical emission spectrometer (ICP/OES) (Varian, 720-ES) was used to determine the concentration of dissolved iron in PA01 media (≥5 ppb). Suspensions of M9 media with particles were spun at 2950 rpm for 15 minutes and filtered with 0.2 µm filters to remove particles before ICP/OES analysis. The concentration of dissolved iron in solution was calculated from the working calibration curve generated from iron standard solution data. Blank samples were also analyzed using ICP/OES to ensure that no significant iron was detected as ions in blank solution. Gamble’s buffer was used for measurement of pH 7.5 experiments and autosomal lysosomal fluid (ALF) was used for pH 4.0 experiments.

### Statistical Analysis

**Figure 1:**
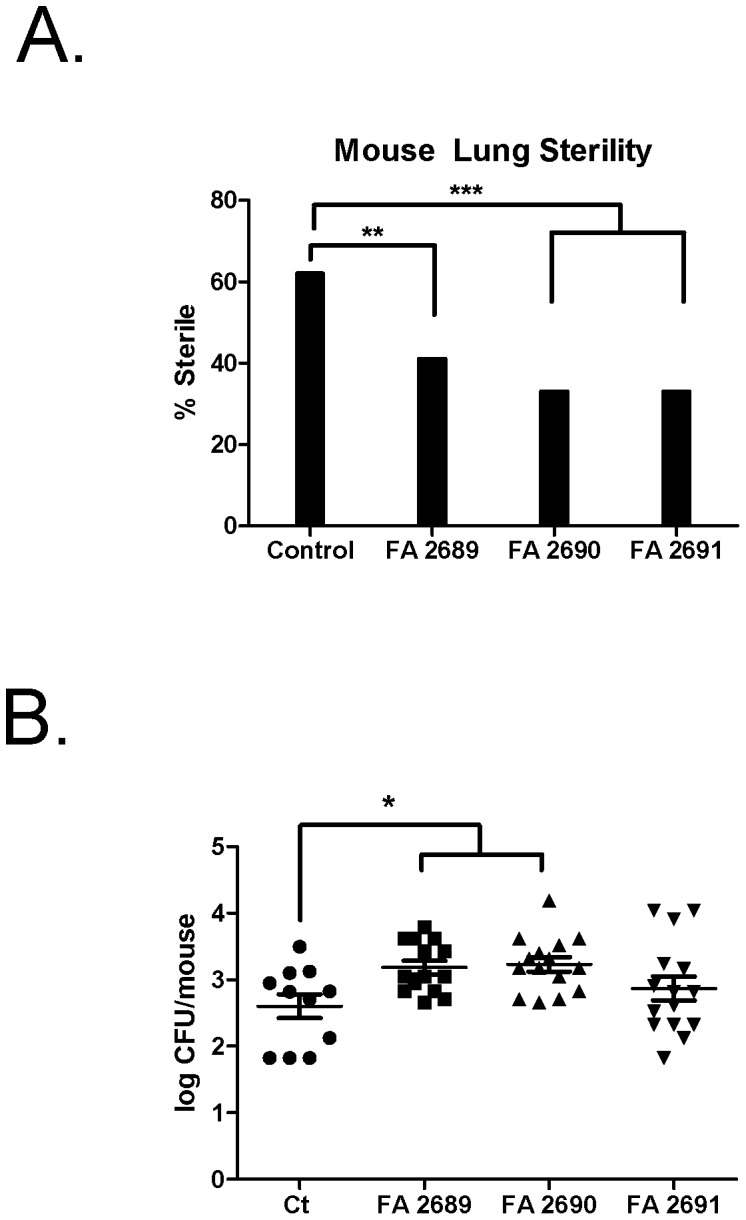
CFA increases bacterial grown *in vivo*. **Panel A.** Total mouse lung sterility *in vivo*. After 24 hours, CFUs were determined after homogenizing lungs and plating to determine growth. In the presence of CFA, lung sterility was decreased by 20–30% **p<0.01, ***p<0.0001. PA01 alone N = 26, PA01+ FA 2689 N = 22, PA01+ FA 2690 N = 21, and PA01+ FA 2691 N = 24. **Panel B.** Growth in non-sterile mice. Log CFU/mouse was determined. FA 2689 and FA 2690 increased growth more than control (p<0.05).

**Figure 2:**
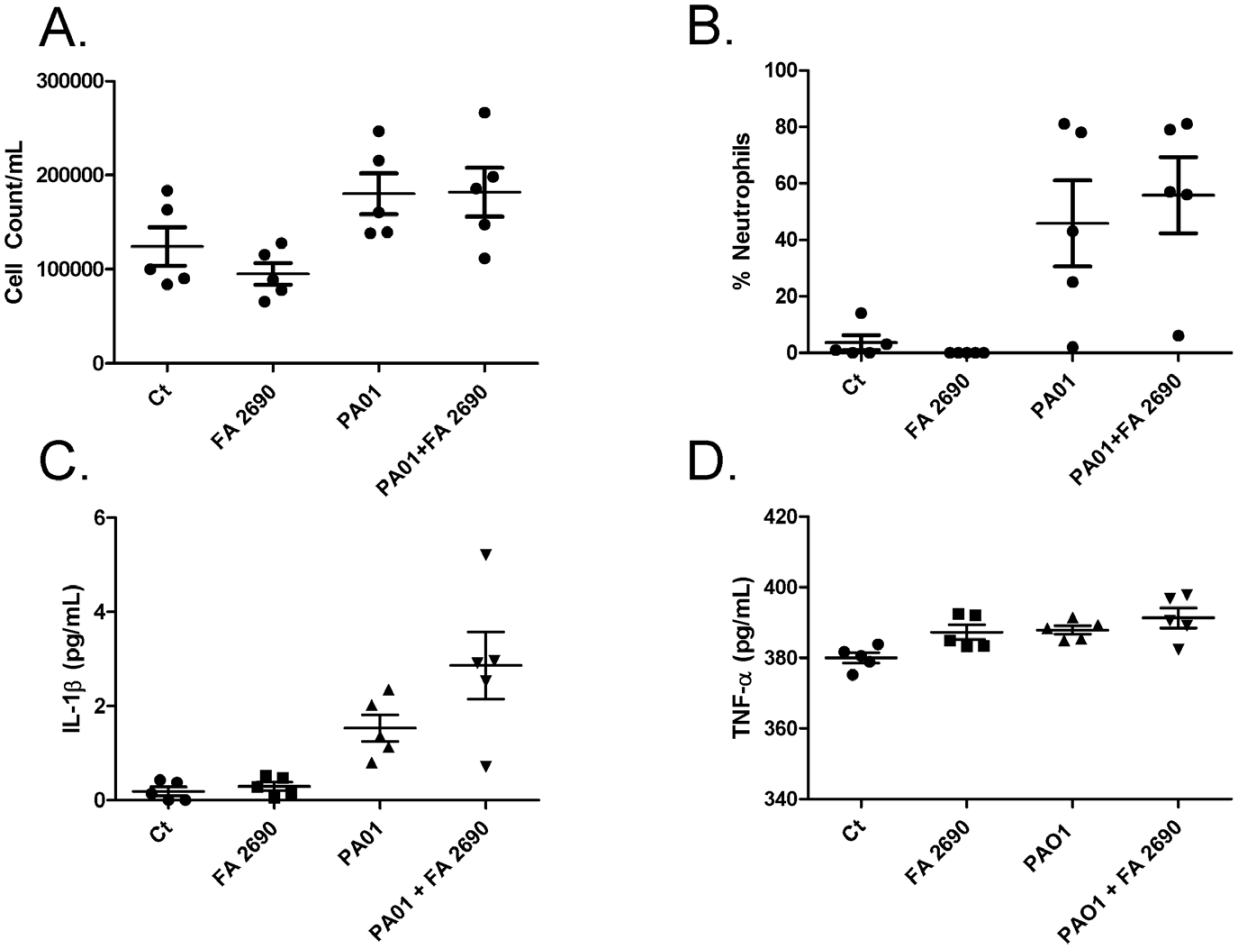
CFA (10 µg/mL) in the presence of PA01 does not significantly increase neutrophil recruitment or cytokine production in BAL. **Panel A.** When C57/Bl6 mice were exposed to 10 µg/mL CFA in the presence and absence of PA01 (4.5 10^6^ PA01/mouse), total BAL cell count was not significantly different between Ct and PA01 but there was an increase in neutrophil percentage in the presence of PA01. However, there were no significant cell count differences between PA01 and PA01 with FA2690. **Panel B.** Although neutrophil recruitment in mice BAL was higher, there was no statistically significant difference between control and PA01 nor PA01 and PA01 with FA2690. **Panel C.** IL-1β in the presence of PA01 and CFA increased production more than PA01 alone but was not significantly different. **Panel D.** TNF-α in the presence of PA01 and CFA does not significantly increase compared with PA01 alone. N = 5.

**Figure 3:**
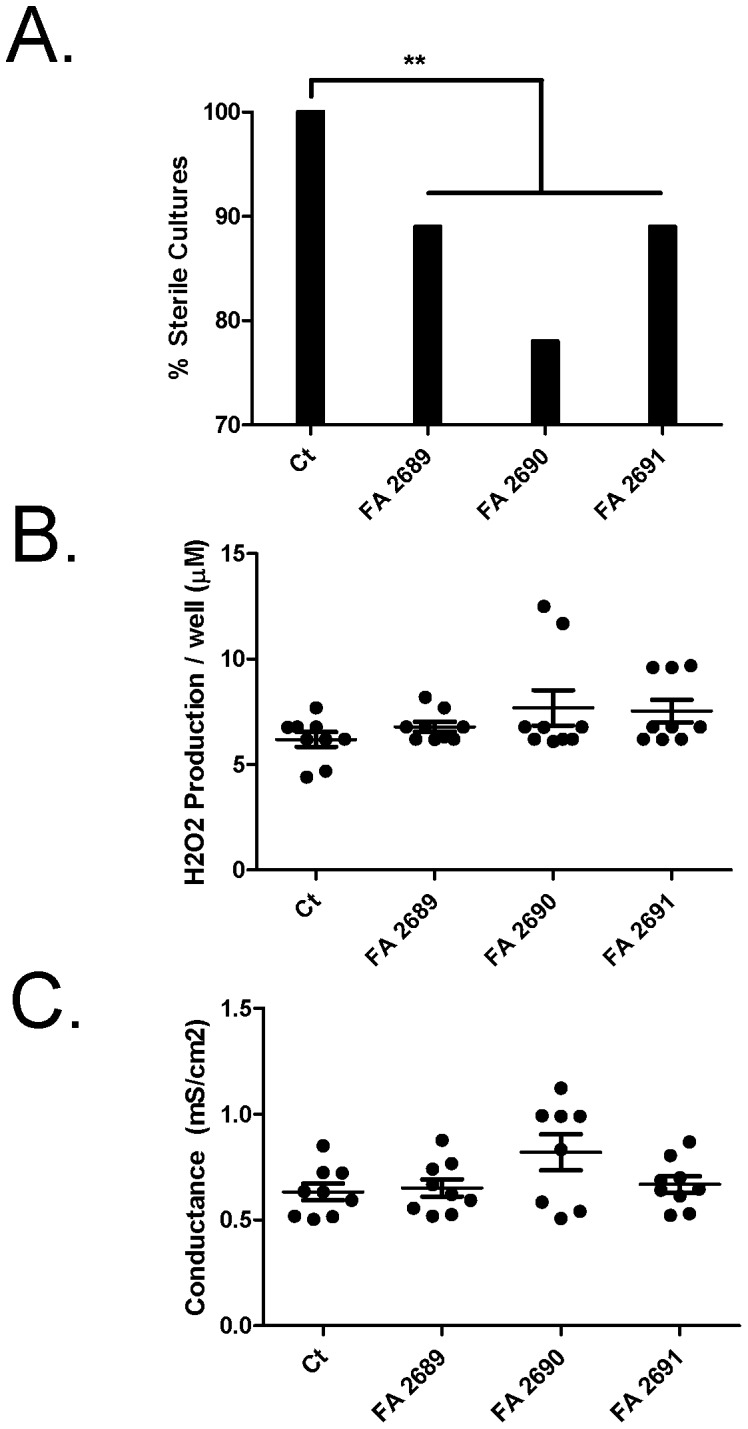
CFA increases PA01 growth in isolated human airway epithelia. **Panel A.** After 24 hours, PA01 (12 CFU) growth in the presence of CFA (10 µg/mL) on isolated human airway epithelia was measured. CFA increases PA01 growth in cell culture. FA 2689 and FA 2691 increased PA01 growth more than CT by 11% and FA 2690 increased PA01 growth 22% **p<0.0001. **Panel B.** Hydrogen peroxide (H_2_O_2_) production in the presence of PA01 and CFA does not significantly increase more than PA01 alone. **Panel C.** Transepithelial electrical conductance (*Gt*) across primary human airway epithelia does not significantly decrease in the presence of PA01 and CFA when compared with PA01 alone. N = 3 in triplicates from two different human donors.

**Figure 4:**
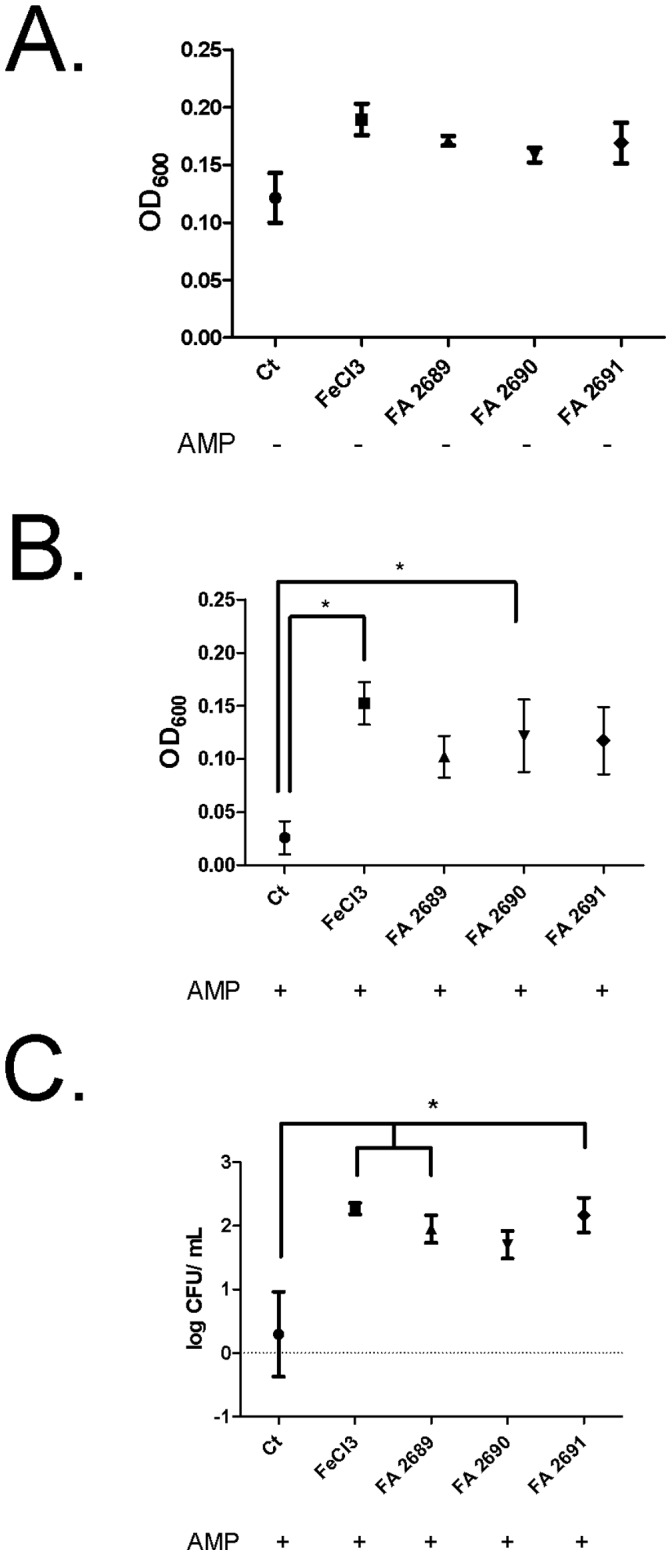
CFA inhibits antimicrobial peptide activity. **Panel A.** PA01 in the presence of FeCl_3_ (25 µM) and three different types of CFA (FA 2689, FA 2690 and FA 2691) increased growth at 10 µg/mL without AMP cocktail more than control, however the growth increase is not statistically significant. **Panel B.** PA01 growth in the presence of AMP cocktail (600 µg/mL Lysozyme, 200 µg/mL Lactoferrin and 100 ng/mL β-Defensin 1&2) inhibits PA01 growth. FeCl_3_ (25 µM) and FA 2690 (10 µg/mL) inhibit AMP activity *p≤0.05, **p<0.0001. **Panel C**. CFU count of PA01 after 18 hours in the presence of AMPs. N = 3 in triplicates. SEM reported.

**Figure 5:**
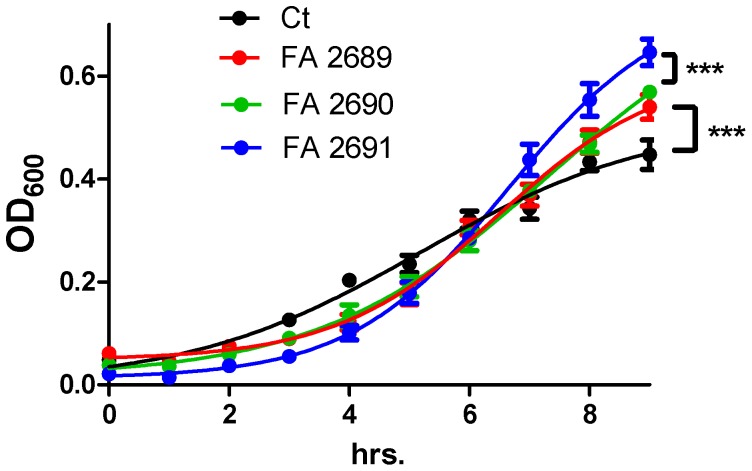
CFA increases PA01 growth. Subcultured PA01 was grown in M9 with FeCl_3_ (25 µM), three different CFA particles (10 µg/mL) or no particles (CT). Growth was recorded over nine hours. CFA increased growth more than CT (p<0.0001 for all three CFAs)_._ FA 2691 increased PA01 growth more than FA 2689 or FA 2690 ***p<0.0001. N = 3 in triplicates.

Data are presented as means ± SEM. The program used for data analysis was GraphPad Prism 5.00 (San Diego, CA). The following information provides the analysis method for each figure and panel. Fisher’s analysis of a contingency table of sterility was used to determine significance in Figure 1A. In Figures 1B and 2A–D, One-way ANOVA using Dunnett’s Multiple Comparison Test was used. Figure 3A, Fisher’s analysis of a contingency table of sterility was used to determine significance. Figures 3B–C and Figures 4A–B, One-way ANOVA using Dunnett’s Multiple Comparison Test was used to determine significance. In Figure 5, non-linear regression (curve-fit) with variable slope from three independent experiments was used for statistical analysis. Data was compared for all parameters of the growth curve using the extra sum of squares F-test to detect differences throughout the entire growth curve. A *p* value of <0.05 was considered statistically significant.

## Results

### CFA Increases PA01 Growth *in vivo*

In order to test our hypothesis of CFA induced PA01 growth, we used a mouse model to determine the effects on PA01 clearance in the presence of CFA. Three CFA particles from different sources that have been well characterized for size, surface area and elemental composition were used in these experiments. Before experiments were conducted, CFA particles were sonicated for ten minutes in order to reduce aggregates. These three sources are standard reference materials (SRM) from the National Institute of Standards and Technology (NIST) and include FA 2689, FA 2690 and FA 2691 (Table 1) [11]. Six to eight week old Harlen C57BL/6 male mice were instilled with PA01 (4.5 10^6^ PA01/mouse) in the presence and absence of CFA (10 µg/mL) in M9 media. According to the ACGIH, insoluble or poorly soluble particles Threshold Limit Value (TLV) is 3 mg/m^3^ for respirable particles and 10 mg/m^3^ for inhalable particles [2]. Therefore, this CFA dose is at the TLV for particles of this composition, which translate our results into relevant daily human exposures.

Twenty four hours later, mice were sacrificed and lungs harvested. None of the mice died over this time period nor were there any significant weight changes (data not shown). After 24 hours, under control conditions, 62% of the mice were sterile. Conversely, FA 2689 only exhibited 41% bacterial clearance (p = 0.0045) and FA 2690 and FA 2691 exhibited 33% bacterial clearance (p<0.0001) (Figure 1A). Among the non-sterile mice, there was a significant amount of bacteria recovered in mice instilled with PA01 and FA 2689 and FA 2690 (p<0.05) (Figure 1B). Therefore, it appears that CFA decreases or delays PA01 clearance in lungs of healthy mice provides a source of iron for bacterial growth or allows the bacteria to persist in the lungs.

### CFA-induced Increased PA01 Recovery is not due to Inflammatory Response *in vivo*

PM has been linked to acute exacerbations of COPD (AECOPD) through neutrophil recruitment and cytokine release [5], [6], [19], [20]. In conjunction with this, air pollution has been shown to inhibit bacterial clearance by increasing inflammation. More specifically, Harrod et al. reported that diesel exhaust particles (DEP) increase infection through an inflammatory response [21]. In order to determine whether the effect of decreased PA01 clearance in our mouse model was due to an increased inflammatory response, we instilled five mice in each condition as stated above and measured neutrophil recruitment and cytokine release. Although the PA01 groups had higher bronchoalveolar lavage (BAL) cell count, the presence of CFA (10 µg/mL, FA2690) did not affect BAL total cell count in the control and PA01 treated group (Figure 2A). In addition, PA01 induced a higher amount of neutrophils (p<0.05) when compared to control, but as shown in Figure 2B, the presence of CFA did not change the neutrophil count significantly more than PA01 alone. After measuring two inflammatory cytokines, TNF-α and IL-1β, it appears that PA01 in the presence of FA 2690 does not increase IL-1β nor TNF-α when compared with PA01 alone (Figures 2C&D).

### CFA on Human Airway Epithelia Increases PA01 Growth without Disrupting Epithelial Barrier Integrity

Since it appears that the decreased airway clearance of PA01 in the presence of CFA *in vivo* is not due to an inflammatory response, we set out to determine if bacterial proliferation in the presence of CFA was due to structural abnormalities at the cellular level. Reactive oxygen species (ROS) production in the airway had been linked to the presence of PM [22] which can generate ROS through oxidation on the surface of the particles, including CFA, which is shown to elicit ROS damage to DNA [23]. It has been reported that in cell culture, at concentrations of 100 µg/mL, CFA with LPS increases ROS [24]. Therefore, using the same cultures for all of the following experiments, we exposed primary human airway epithelial cells cultured in an air-liquid interface [16] to PA01 (12 CFU in 0.1 µL) in the presence of 10 µg/mL CFA and tested growth, trans-epithelial electrical conductance (*Gt*) and hydrogen peroxide (H_2_O_2_) production. As shown in Figure 3A, human airway epithelial cells were 100% sterile after 24 hours of incubation with PA01, while the presence of CFA impaired airway bacterial clearance or increase bacterial growth (p<0.0001). Specifically, the percentage of growth in cell cultures treated with FA 2689 and FA 2691 was 11% (95% CI 0 to 37%) whereas in the presence of FA 2690, growth was present in 22% (95% CI 0 to 56%) of the cultures (n = 3 in triplicates from two different human donors).

After 24 hours, H_2_O_2_ production was measured by determining p-hydroxyphenylacetate (pHPA) oxidation [25]. CFA (10 µg/ml) in the presence of PA01 does not increase H_2_O_2_ production when compared with PA01 alone, which is consistent with other reports [24] (Figure 3B). Also, *Gt* was measured to determine the effect on the epithelial barrier integrity. Exposing primary human airway epithelial cells to CFA and PA01 at the above concentrations did not disrupt airway epithelial barrier integrity (Figure 3C), nor increased cell death as determined by propidium iodide staining (data not shown).

### CFA Decreases Antimicrobial Peptide Activity

The above results led to the hypothesis that CFA impairs airway innate immunity mechanisms. The lung has various mechanisms to protect itself against pathogens and one of the primary defense systems are AMPs, which are present in the airway surface liquid (ASL). This is comprised primarily of lactoferrin, lysozyme and β-Defensins 1&2, which behave synergistically but also have specific functions. Specifically, lysozyme degrades the bacterial cell walls via its muramidase activity, lactoferrin sequesters iron and inhibits microbial respiration, therefore limiting iron availability, and β-defensins have broad antibacterial activity [26].

In order to test whether CFA inhibits AMP activity, we combined physiologically relevant concentrations of AMP present in the lung [27] (600 µg/mL Lysozyme, 200 µg/mL Lactoferrin and 100 ng/mL B Defensins 1&2) with 10 µg/mL CFA and determined the effect on PA01 growth by measuring OD_600_ after 18 hours. As shown in Figure 4A, PA01 grew in the absence of AMP. In the presence of a positive control, FeCl_3_ (25 µM), and CFA (10 µg/mL) there was an increase in bacterial growth, although not statistically significant. When PA01 was treated with AMP, there was significant growth inhibition. Conversely, as shown in Figures 4B & C, AMP activity was impaired when PA01 cultures (determined by measuring CFU and OD_600_) were treated with all three forms of CFA and FeCl_3_.

### CFA Provides a Bioavailable Source of Iron for PA01

Due to the increase in bacterial growth (Figure 4A), we set out to determine if there were growth differences between the three different CFA particles. Before conducting our experiments, we set out to create a media with non-detectable iron levels (<5 ppb; M9) in order to mimic as much as possible the ASL iron content. To test our hypothesis of CFA induced bacteria growth and the role of dissolved iron, 10 µg/mL of CFA particles (FA 2689, 2690, 2691) were added to three hour sub-cultured PA01 cultures in M9 media and growth was observed by measuring OD_600_ at 37°C for nine hours while correcting for particle absorbance effects. FeCl_3_ (25 µM), a soluble source of iron, was used as a positive control (data not shown). All CFA particles induced bacteria growth compared to control (p<0.0001). In addition, FA 2691 appeared to contribute to PA01growth more than FA 2689 and FA 2690 (Figure 5) (p<0.0001). When iron dissolution in M9 media was measured, using an inductively coupled plasma optical emission spectrometer (ICP-OES), FA 2691 had 0.057 mg/L of dissolved iron compared with 0.028 and 0.032 mg/L in FA 2689 and 2690 respectively (Table 1).

## Discussion

The World Health Organization (WHO) reports that acute respiratory infections (ARIs) are the leading cause of acute illnesses worldwide and remain one of the most important causes of death, especially in the very young, the elderly, and the immunocompromised. In addition, the WHO and the Environmental Protection Agency (EPA) recognize ambient air pollution as an important risk factor for ARIs. Despite the magnitude of this problem, the ambient air pollution mechanism responsible for the development of respiratory infections is not well known. One of the main components of ambient air pollution is particulate matter (PM), thus PM must play an important role in this mechanism. Since respiratory infections are in part the consequence of mechanisms that will promote bacterial growth and will impair innate immunity, we initially hypothesize that PM will increase nutrient bioavailability for bacteria, and will impair airway antimicrobial peptide function.

Our results show that CFA reduces or delays bacterial clearance *in vivo* and *in vitro* as well as provides a source of iron for bacterial growth. The reduced bacterial clearance is consistent with reports of rats and mice exposed to PM [21], [28], where one mechanism implicated in bacterial clearance impairment is an increase inflammatory response in the lung. However, the overall inflammatory response in the presence of CFA and PA01 was not significantly increased over PA01 alone. This discrepancy when compared with previous studies reporting a correlation between neutrophil recruitment and increased infection is perhaps due to differences in dose, 50 µg reported versus 500 ng in our study [29], [30]. Another potential mechanism of reduced bacterial clearance could be due to macrophage function impairment. This mechanism has been shown in models where PM exposure, at high doses, inhibits phagocytosis. However, the relevance of this inhibition in people exposed to ambient air pollution has been raised recently in a review by Miyata et al. [31]. Part of the argument lies in regard to the experimental doses that show this effect, and its relevance to actual ambient air pollution exposures. In contrast, our *in vivo* and *in vitro* models, with much lower PM doses, suggest other mechanisms of increased infection susceptibility, specifically the impairment of AMP function. Our results of reduced AMP activity in the presence of CFA provide insight into diseases with persistent colonization, which is consistent with a recent report that showed AMP activity impairment in a cystic fibrosis model [32]. Also, Parameswaran et al. reported that AMP levels in COPD subjects likely affect pathogen clearance and clinical outcomes of infection [33].

Determining the mechanism of CFA impairment on AMP function is challenging since these particles are physicochemically complex. However, it seems that PM could potentially decrease lysozyme activity. For example, a small cohort of peat dust exposed workers showed decreased lysozyme positive macrophages, indicating increased macrophage phagocytosis and a potential effect on lysozyme activity [34]. Also Noble et al. reported that cigarette smoke and dust decreased human nasal lysozyme concentrations [35]. Other studies that have attempted to understand the mechanism of lysozyme inhibition has shown that lysozyme activity is inhibited by cations [36] thus CFA particles could leach certain cations, such as Fe (III), Fe (II) or Al (III) and inhibit AMP activity. PM not only can affect lysozyme, but β-Defensins, as it has been reported that oil fly ash, a byproduct of oil-fired power plants with a composition of carbon, silicates, and iron oxides can impair β-Defensin synthesis in epithelial cells [37]. Furthermore, lactoferrin can be inhibited by its complete iron saturation, which in turns, impairs it ability to sequester iron. Therefore, several mechanisms impairing AMP function can potentially play a role in PM induced decrease bacterial clearance.

CFA not only decreased AMP activity, but increased bacterial growth. CFA is known to be an iron containing particle, thus CFA can be an important nutrient for bacteria growth. In addition, a recent report correlated iron mobilization in CFA with iron associated within aluminosilicate glass phases [13]. Therefore, it appears the effect of CFA on bacterial growth is more complex than just total iron content alone, since FA 2689 had the largest amount of iron, but it did not translate into the highest dissolved iron [11], nor in the highest growth curve. As shown in Table 1, the propensity for iron to be mobilized and thus dissolved is due in part to its enrichment within the aluminum silicate phase, specifically iron in FA 2691 is to a large extent associated with the aluminum silicate content (XPS/EDX ratio range: 0.4–0.8 for Al, Fe and Si) (Table 1) compared with FA 2689 (XPS/EDX ratio range: 0.6–1.3) and FA 2690 (XPS/EDX ratio range: 0.6–1.4). CFA spheres commonly contain aluminosilicate-phase iron in the inner core with iron oxide aggregate on the surface (see Chen, Laskin et al. 2012) Therefore, due to the decreased XPS/EDX ratio and thus high iron content in aluminosilicate phase, we propose that one mechanism of PM induced bacteria growth is dependent on the iron dissolution from the aluminum silicate glass content (Table 1).

In summary, our results show the following: i) the CFA concentrations used in this study are potentially very relevant to human daily exposures, thus posing a potential public health risk for susceptible subjects living in urban areas and for those exposed to Fe-containing anthropogenic particles; ii) although CFA provides a source of bioavailable iron for bacteria, not all CFA particles have the same biological effects, and their propensity for iron dissolution can be an important factor on susceptible subjects and populations; iii) CFA impairs lung innate immune mechanisms of bacterial clearance, specifically AMP activity.

These results provide a potential mechanism to explain the epidemiological data that associates ambient air pollution and bacterial infections. However, we recognize that PM is very complex and requires the design of experiments that will control for different physicochemical characteristics such as size, shape, presence of other transition metals, aluminum silicate content, and iron species. We expect that identifying the PM mechanisms of respiratory infections will translate into public health policies aimed at controlling, not only concentration of PM exposure, but physicochemical characteristics that will potentially cause respiratory infections in susceptible individuals and populations.

## References

[1] Watson W, Paduano N, Raghuveer T, Thapa S (2010) U.S. Coal Supply and Demand: 2010 Year in Review. In: Administration USEI, editor. Washington, DC: Environmental Protection Agency.

[2] Hygienists ACGIH (2009) The Documentation of the Threshold Limit Values and Biological Exposure Indicies. Cincinnati: ACGIH. 74–75.

[3] Giere R, Carleton LE, Lumpkin GR (2003) Micro- and nanochemistry of fly ash from a coal-fired power plant. American Mineralogist 88: 1853–1865.

[4] Goss CH, Newsom SA, Schildcrout JS, Sheppard L, Kaufman JD (2004) Effect of ambient air pollution on pulmonary exacerbations and lung function in cystic fibrosis. American journal of respiratory and critical care medicine 169: 816–821. 14718248 10.1164/rccm.200306-779OC

[5] Ling SH, Van Eeden SF (2009) Particulate matter air pollution exposure: role in the development and exacerbation of chronic obstructive pulmonary disease. Int J Chron Obstruct Pulmon Dis 4: 233–243. 19554194 10.2147/copd.s5098 PMC2699820

[6] Arbex MA, de Souza Conceicao GM, Cendon SP, Arbex FF, Lopes AC, et al (2009) Urban air pollution and chronic obstructive pulmonary disease-related emergency department visits. Journal of epidemiology and community health 63: 777–783. 19468016 10.1136/jech.2008.078360

[7] Gilligan PH (1991) Microbiology of airway disease in patients with cystic fibrosis. Clinical microbiology reviews 4: 35–51. 1900735 10.1128/cmr.4.1.35 PMC358177

[8] Smith KR, Samet JM, Romieu I, Bruce N (2000) Indoor air pollution in developing countries and acute lower respiratory infections in children. Thorax 55: 518–532. 10817802 10.1136/thorax.55.6.518 PMC1745777

[9] Ghio AJ, Carraway MS, Madden MC (2012) Composition of air pollution particles and oxidative stress in cells, tissues, and living systems. Journal of toxicology and environmental health Part B, Critical reviews 15: 1–21. 10.1080/10937404.2012.632359 22202227

[10] Shi Z, Krom MD, Bonneville S, Baker AR, Bristow C, et al. (2011) Influence of chemical weathering and aging of iron oxides on the potential iron solubility of Saharan dust during simulated atmospheric processing. Global Biogeochemical Cycles 25.

[11] Chen H, Laskin A, Baltrusaitis J, Gorski CA, Scherer MM, et al (2012) Coal Combustion Fly Ash as a Source of Iron in Atmospheric Dust. Environmental science & technology 46(4): 2112–2120. 22260270 10.1021/es204102f

[12] Veranth JM, Smith KR, Hu AA, Lighty JS, Aust AE (2000) Mobilization of iron from coal fly ash was dependent upon the particle size and source of coal: analysis of rates and mechanisms. Chemical research in toxicology 13: 382–389. 10813655 10.1021/tx9901884

[13] Veranth JM, Smith KR, Huggins F, Hu AA, Lighty JS, et al (2000) Mossbauer spectroscopy indicates that iron in an aluminosilicate glass phase is the source of the bioavailable iron from coal fly ash. Chemical research in toxicology 13: 161–164. 10725111 10.1021/tx9902136

[14] Bullen JJ, Rogers HJ, Spalding PB, Ward CG (2005) Iron and infection: the heart of the matter. FEMS immunology and medical microbiology 43: 325–330. 15708305 10.1016/j.femsim.2004.11.010

[15] Baltrusaitis J, Usher CR, Grassian VH (2007) Reactions of sulfur dioxide on calcium carbonate single crystal and particle surfaces at the adsorbed water carbonate interface. Physical chemistry chemical physics : PCCP 9: 3011–3024. 17551626 10.1039/b617697f

[16] Karp PH, Moninger TO, Weber SP, Nesselhauf TS, Launspach JL, et al (2002) An In Vitro Model of Differentiated Human Airway Epithelia: Epithelial Cell Culture Protocols. Methods in molecular biology 188: 115–137. 11987537 10.1385/1-59259-185-X:115

[17] Caraballo JC, Yshii C, Butti ML, Westphal W, Borcherding JA, et al (2011) Hypoxia increases transepithelial electrical conductance and reduces occludin at the plasma membrane in alveolar epithelial cells via PKC-zeta and PP2A pathway. American journal of physiology Lung cellular and molecular physiology 300: L569–578. 21257729 10.1152/ajplung.00109.2010 PMC3075095

[18] Kaneko Y, Thoendel M, Olakanmi O, Britigan BE, Singh PK (2007) The transition metal gallium disrupts Pseudomonas aeruginosa iron metabolism and has antimicrobial and antibiofilm activity. The Journal of clinical investigation 117: 877–888. 17364024 10.1172/JCI30783 PMC1810576

[19] Finnerty K, Choi JE, Lau A, Davis-Gorman G, Diven C, et al (2007) Instillation of coarse ash particulate matter and lipopolysaccharide produces a systemic inflammatory response in mice. Journal of toxicology and environmental health Part A 70: 1957–1966. 17966067 10.1080/15287390701549229

[20] Nurkiewicz TR, Porter DW, Barger M, Millecchia L, Rao KM, et al (2006) Systemic microvascular dysfunction and inflammation after pulmonary particulate matter exposure. Environmental health perspectives 114: 412–419. 16507465 10.1289/ehp.8413 PMC1392236

[21] Harrod KS, Jaramillo RJ, Berger JA, Gigliotti AP, Seilkop SK, et al (2005) Inhaled diesel engine emissions reduce bacterial clearance and exacerbate lung disease to Pseudomonas aeruginosa infection in vivo. Toxicological sciences : an official journal of the Society of Toxicology 83: 155–165. 15483187 10.1093/toxsci/kfi007

[22] Mukherjee B, Dutta A, Roychoudhury S, Ray MR (2011) Chronic inhalation of biomass smoke is associated with DNA damage in airway cells: involvement of particulate pollutants and benzene. Journal of applied toxicology 33(4): 281–289. 22131134 10.1002/jat.1748

[23] Jones T, Brown P, BeruBe K, Wlodarczyk A, Longyi S (2010) The physicochemistry and toxicology of CFA particles. Journal of toxicology and environmental health Part A 73: 341–354. 20155577 10.1080/15287390903442637

[24] Voelkel K, Krug HF, Diabate S (2003) Formation of reactive oxygen species in rat epithelial cells upon stimulation with fly ash. Journal of biosciences 28: 51–55. 12682424 10.1007/BF02970131

[25] Panush D, Fulbright R, Sze G, Smith RC, Constable RT (1993) Inversion-recovery fast spin-echo MR imaging: efficacy in the evaluation of head and neck lesions. Radiology 187: 421–426. 8475284 10.1148/radiology.187.2.8475284

[26] Wiesner J, Vilcinskas A (2010) Antimicrobial peptides: the ancient arm of the human immune system. Virulence 1: 440–464. 21178486 10.4161/viru.1.5.12983

[27] Ganz T (2002) Antimicrobial polypeptides in host defense of the respiratory tract. The Journal of clinical investigation 109: 693–697. 11901174 10.1172/JCI15218 PMC150915

[28] Roberts JR, Young SH, Castranova V, Antonini JM (2009) The soluble nickel component of residual oil fly ash alters pulmonary host defense in rats. Journal of immunotoxicology 6: 49–61. 19519163 10.1080/15476910802630379

[29] Sigaud S, Goldsmith CA, Zhou H, Yang Z, Fedulov A, et al (2007) Air pollution particles diminish bacterial clearance in the primed lungs of mice. Toxicology and applied pharmacology 223: 1–9. 17561223 10.1016/j.taap.2007.04.014 PMC2075081

[30] Hatch GE, Boykin E, Graham JA, Lewtas J, Pott F, et al (1985) Inhalable particles and pulmonary host defense: in vivo and in vitro effects of ambient air and combustion particles. Environmental research 36: 67–80. 3967645 10.1016/0013-9351(85)90008-8

[31] Miyata R, van Eeden SF (2011) The innate and adaptive immune response induced by alveolar macrophages exposed to ambient particulate matter. Toxicology and applied pharmacology 257: 209–226. 21951342 10.1016/j.taap.2011.09.007

[32] Pezzulo AA, Tang XX, Hoegger MJ, Alaiwa MH, Ramachandran S, et al (2012) Reduced airway surface pH impairs bacterial killing in the porcine cystic fibrosis lung. Nature 487: 109–113. 22763554 10.1038/nature11130 PMC3390761

[33] Parameswaran GI, Sethi S, Murphy TF (2011) Effects of bacterial infection on airway antimicrobial peptides and proteins in COPD. Chest 140: 611–617. 21349930 10.1378/chest.10-2760 PMC3204796

[34] Sandstrom T, Kolmodin-Hedman B, Ledin MC, Bjermer L, Hornqvist-Bylund S, et al (1991) Exposure to peat dust: acute effects on lung function and content of bronchoalveolar lavage fluid. British journal of industrial medicine 48: 771–775. 1954154 10.1136/oem.48.11.771 PMC1035452

[35] Noble RE (2002) Effect of environmental contaminants on nasal lysozyme secretions. The Science of the total environment 284: 263–266. 11846170 10.1016/s0048-9697(01)00871-3

[36] Travis SM, Conway BA, Zabner J, Smith JJ, Anderson NN, et al (1999) Activity of abundant antimicrobials of the human airway. American journal of respiratory cell and molecular biology 20: 872–879. 10226057 10.1165/ajrcmb.20.5.3572

[37] Klein-Patel ME, Diamond G, Boniotto M, Saad S, Ryan LK (2006) Inhibition of beta-defensin gene expression in airway epithelial cells by low doses of residual oil fly ash is mediated by vanadium. Toxicological sciences : an official journal of the Society of Toxicology 92: 115–125. 16641320 10.1093/toxsci/kfj214 PMC2147678

